# Supplementary material for: Causal Relationship between Aging and Anorexia Nervosa: A White-Matter-Microstructure-Mediated Mendelian Randomization Analysis
Source: Biomedicines. 2024 Aug 16;12(8):1874. doi: 10.3390/biomedicines12081874 (PMC11351342; doi:10.3390/biomedicines12081874)
Supplement: Supplementary file 1 [file biomedicines-12-01874-s001.zip › Supplementary Figure/Figure S8/Leaveoneout plot of MR analysis for TL to ubm-b-2029.pdf]

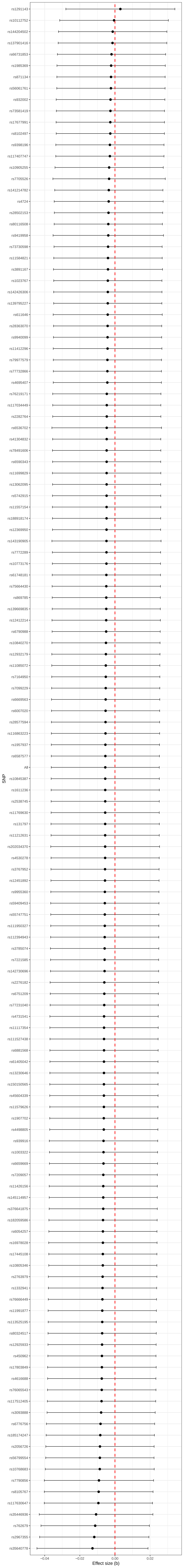

SNP
Effect size (b)
-0.04
-0.02
0.00
0.02
rs1291143
rs10112752
rs144204502
rs137901416
rs66731853
rs1985369
rs871134
rs56061761
rs932002
rs73581419
rs17677991
rs8102497
rs9398196
rs117407747
rs10905255
rs7705526
rs141214782
rs4724
rs28502153
rs80116508
rs9419958
rs73730598
rs11584821
rs3891167
rs1023767
rs142426306
rs139795227
rs611646
rs28363070
rs9940099
rs11412296
rs79977579
rs77732866
rs4695407
rs76219171
rs117034449
rs2282764
rs6536702
rs41304832
rs78491606
rs6590343
rs11699829
rs13062095
rs5742915
rs11557154
rs188918174
rs12369950
rs143190905
rs7772289
rs10773176
rs61748181
rs75664430
rs869785
rs139669835
rs12412214
rs6790988
rs10840270
rs12932178
rs11085072
rs7164950
rs7099229
rs6669563
rs6007020
rs28577594
rs116863223
rs1957937
rs6587577
All
rs10845387
rs1611236
rs2538745
rs11769630
rs131797
rs11212631
rs202034370
rs4530278
rs3767952
rs12451892
rs9955360
rs59409453
rs55747751
rs111950327
rs112394943
rs3785074
rs7221585
rs142730696
rs2276182
rs6751209
rs77231040
rs4731541
rs11117354
rs111527438
rs6881568
rs61405042
rs13230646
rs150150565
rs45604339
rs11579626
rs1907702
rs4498805
rs939916
rs1003322
rs6659669
rs7209057
rs11426156
rs145114957
rs376641875
rs182059586
rs6054257
rs16978028
rs17445108
rs10805346
rs2763979
rs1332941
rs76666449
rs11991877
rs113525195
rs80324517
rs12925933
rs450962
rs17803849
rs4616688
rs76065543
rs117512405
rs3093888
rs6776756
rs185174247
rs2056726
rs56799554
rs10768683
rs7790856
rs8105767
rs117630647
rs35446936
rs762679
rs2967355
rs35640778
